# Views and Experiences of LGBTQ+ People in Prison Regarding Their Psychosocial Needs: A Systematic Review of the Qualitative Research Evidence

**DOI:** 10.3390/ijerph18179335

**Published:** 2021-09-03

**Authors:** Gráinne Donohue, Edward McCann, Michael Brown

**Affiliations:** 1 School of Nursing and Midwifery, Trinity College Dublin, Dublin, Ireland; mccanned@tcd.ie; 2 School of Nursing and Midwifery, Queen’s University, Belfast BT9 7BL, UK; m.j.brown@qub.ac.uk

**Keywords:** prisons, LGBTQ, mental health, psychosocial, supports, experiences, qualitative evidence, systematic review

## Abstract

People who identify as LGBTQ+ and are in prison often experience many additional challenges. Once in prison, there is systemic discrimination against imprisoned LGBTQ+ people and a lack of understanding and concern regarding their care, treatment and support needs. While there is growing interest in their protection and that of other vulnerable populations in prison settings, little is known about their views and experiences regarding their distinct psychosocial needs. The aim of this systematic review is to critically evaluate and synthesize the existing research evidence relating to the unique psychological and social experiences of LGBTQ+ people in prison and identify aspects that may help or hinder access to appropriate psychosocial interventions and supports. The PRISMA procedure was utilized. A search of relevant databases from January 2010 to March 2021 was undertaken. Studies were identified that involved LGBTQ+ people, and addressed their views and experiences regarding their psychosocial needs whilst in prison. The search yielded 858 papers in total. Following the application of rigorous inclusion and exclusion criteria a total of 12 papers were considered suitable for the systematic review. Quality was assessed using the CASP instrument. Following analysis, three themes were identified: (i) interpersonal factors (ii) intrapersonal factors and (iii) institutional factors. The policy, education and practice development implications are highlighted and discussed. Future research opportunities have been identified that will add significantly to the body of evidence that may further the development of appropriate health interventions and supports specific to the LGBTQ+ population in prison.

## 1. Introduction

It is now well established that LGBTQ+ people experience a higher prevalence of mental ill-health than the general population, with poorer mental health outcomes [1]. For transgender people specifically, this relates to higher levels of depression, suicidality, interpersonal trauma, substance use disorders and general distress [2]. LGBTQ+ people who are in prison experience a number of additional challenges. These factors are not singular, they are interlocking, giving rise to a vast inequality in the well-being of those in prisons compared to their heterosexual or cisgender counterparts [3]. Amongst the factors of concern are institutional stigma buoyed by pervasive myths and stereotypes about the population. For example, research has shown that the discrimination, alienation and victimization that LGBTQ+ people can experience in general society is often mirrored and intensified in the prison environment [4]. However, this stigma can also inform institutional culture and behavior toward inmates and may reinforce lack of agency amongst prison staff regarding how to work and support this population. The United Nations Office on Drugs and Crime (UNODC) [5] has added to research by recognizing issues of heteronormativity, homophobia and transphobia both within and outside prison. Within prison specifically is the threat of physical and sexual violence, institutional discrimination and neglect, unmet health needs and social isolation.

Despite the decriminalization of homosexuality across most regions, the historical legacy of incarceration amongst this population has not diminished. There are strong indications that LGBTQ+ people are still more likely to become involved in the criminal justice system than the heterosexual or cisgender community [6]. A National LGBTQ+ Prisoner Survey published in 2015, found that close to two thirds (58%) of respondents’ first arrest occurred when they were under the age of 18 [3]. This same report details LGBTQ+ people, particularly of color and coming from poverty, experience high levels of policing and criminalization, leading to arrest and incarceration. Reasons for this high incidence include the experiences of discrimination and harassment among LGBTQ+ young people that may lead to higher levels of engagement in risky and illicit behaviours, resulting in increased contact with criminal justice authorities [7,8]. Whilst quite strong evidence exists for the over-representation of LGBTQ+ juveniles in the US [6], there is limited international evidence to support this and mixed evidence for adult LGB populations. There is consistent evidence however for the over-representation of trans women/trans feminine population in prison and some evidence on trans men/trans masculine populations [9]. Most consistently, surveys of prisoners show far lower rates of self-declared LGB prisoners in men’s prisons and higher in women’s [10].

Sexual and gender minority people who are incarcerated experience high rates of sexual victimization in U.S. prisons as compared to other inmates [4]. Transgender people specifically pose a set of unique challenges to the prison environment. These challenges include breach of rules about dress, the risk of sexual, physical and emotional victimization from other inmates and staff in prison and frequently, inadequate access to appropriate health care [11]. Jenness et al. compared a sample of 39 transgender women prisoners and a random sample of 322 male prisoners and found that 59% of transgender women reported experiencing sexual assault in prison compared to 4% of the random sample [12]. In the USA, studies indicate that transgender people in prison do not receive adequate physical and mental healthcare provision and that there has been an inadequate response to these issues amongst policy makers [13,14]. In 2003, the U.S. Congress passed the Prison Rape Elimination Act [15], which aimed to establish a zero-tolerance policy toward prison rape and minimize sexual violence in correctional facilities. Since then, sexual violence in prison has received more attention from researchers and the public.

Since the Yogyakarta Principles were adopted in 2006, they have developed into an authoritative statement of the human rights of persons of diverse sexual orientations and gender identities [16]. Jogyakata Principle 9 relates to the right to a humane treatment while in detention. Amongst the obligations emphasized, is the importance of independent monitoring of detention facilities by the State in the areas of sexual orientation and gender identity. The National Institute of Corrections in the USA (2015) undertook a substantive review of constitutional law, recommending the development and implementation of LGBT-affirmative policies [17].

There is a growing movement to protect LGBTQ+ people in prison and other vulnerable incarcerated populations. However, in order for the relevant policies and procedures to be implemented, it is necessary to understand and define the psychosocial views and experiences of incarcerated LGBTQ+ people. Therefore, the aim of this review is to systematically evaluate the best available research evidence of the views and experiences of LGBTQ+ people regarding their psychosocial needs in prison and to identify best practice that may enable access to necessary psychosocial interventions and supports.

## 2. Methods

The objectives of the systematic review were:

1. To synthesize the best available qualitative evidence regarding the views and experiences LGBTQ+ people in prisons and their psychosocial needs;
2. To identify psychosocial interventions and supports of LGBTQ+ people in prisons;
3. To highlight areas of good practice regarding meeting the psychosocial needs of LGBTQ+ people in prisons

### 2.1. Ethics Statement

The study is a systematic review of published research evidence therefore ethical approval was not required.

### 2.2. Search Strategy

A subject Librarian was enlisted to assist with the literature search strategy. The databases used in the search were CINHAL (EBSCO host), MEDLINE (Ovid) and PsycINFO (Ovid). The search strategy used in all of the electronic databases is shown in Table 1. Databases were searched up until 31st March 2021. Considered papers were written in English, peer reviewed and published after 2010. A hand search was conducted of reference lists and Google Scholar used to identify any other relevant papers.

### 2.3. Inclusion and Exclusion Criteria

Included studies had used a qualitative approach and addressed the distinct experiences and perceptions of LGBTQ+ people who are incarcerated in prison. Studies that identified psychosocial issues and supports were considered for inclusion. Psychosocial issues or supports in this context refers to phenomena that can affect a person’s functioning in daily life, his or her environment and/or life events. In the prison setting, this can refer to psychological concerns including mental health issues or social problems such as housing/accommodation, lack or poor access to adequate healthcare, social-cultural problems, legal problems, adjustment problems or issues around loss and identity. Studies that did not specifically focus on the inmate experience or deal with the objectives of the review were excluded.

### 2.4. Data Extraction

Following the removal of duplicate papers, two reviewers screened the title and abstracts using the study inclusion criteria. The Covidence systematic review software (Veritas Health Innovation, Melbourne, Australia) was used in the process [18]. The reviewers retrieved and independently screened the full text papers. The reviewers then critically appraised the papers, and any disagreements were resolved following discussion.

### 2.5. Data Synthesis

The synthesis of the research literature was carried out as part of the systematic review process. The data was thematically analyzed to identify the emergent themes across the included studies. Detailed and comprehensive coding and analysis was undertaken. The identified concepts were grouped into themes to enable comparisons and differences to be established across and between the studies and the themes. The final themes were discussed, verified and agreed by the research team (EM, GD and MB).

### 2.6. Quality Assessment

The included papers were appraised for methodological quality. A well-recognized and commonly used critical appraisal instrument was utilized to assess the quality of the selected papers (Critical Appraisal Skills Programme) [19]. Ten questions in total were applied to the data (Table 2). Each item was rated ‘yes’, ‘no’, and ‘can’t tell’. A ‘yes’ response meant that a clear statement appeared in the paper that directly answered the question. ‘No’, indicated that the question was answered negatively in the paper, and ‘Can’t tell’, indicated that there was no clear statement relating to the question. There was a high amount of ‘yes’ responses across most of the papers for a significant number of questions indicating good quality overall. Question number 6 had particularly low ratings indicating that the ‘research relationships’ were either not considered or unable to tell if this was so. Any disagreements were discussed and consensus reached. All of the studies addressed the objectives of the review and were considered suitable for inclusion in the systematic review.

## 3. Results

### 3.1. Search Results

**Figure 1:**
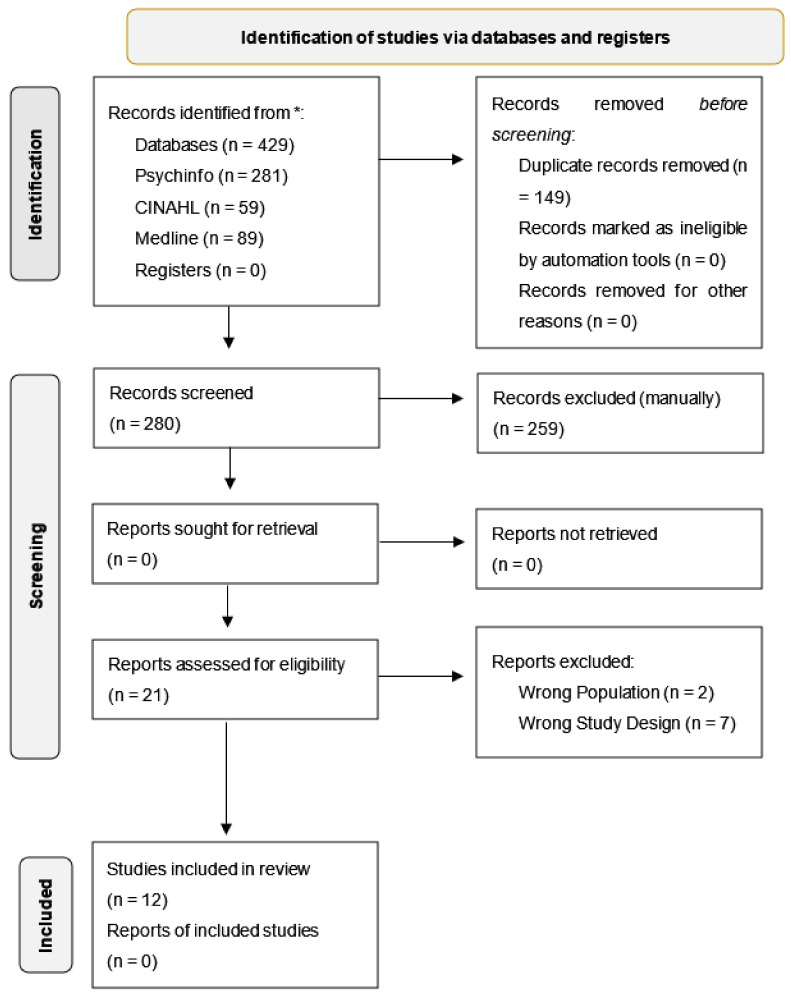
Prisma.

The Preferred Reporting Items for Systematic Reviews and Meta-analyses Statement (PRISMA-S) process was followed, and a flow chart is provided (Figure 1) that contains the results of the searches [20]. Initial searches revealed 429 hits across all the databases. Search limiters of peer reviewed empirical studies and written in English were applied and duplicates were removed leaving 280 papers for abstract screening. Following this step, a total of 21 full text papers were assessed for eligibility using the specified inclusion criteria. A hand search was also conducted of the reference lists of the identified papers leaving a total of 12 papers for the review [21,22,23,24,25,26,27,28,29,30,31,32]. In line with PRISMA guidelines, this review has been registered with PROSPERO.

### 3.2. Study Characteristics

The 12 studies that addressed the aim and objectives of the review are contained in Table 3. The majority of studies (n = 9) were undertaken in the USA [21,22,24,25,26,27,30,31,32]. The remaining studies were from Australia (n = 2) [23,28] and Brazil/Italy (n = 1) [29]. Study participant numbers ranged from 7 to 315. The age range of participants across studies was 19–65 years. The institutional settings included in these reviews were varied and in the main were based on multiple site settings, which included one city-wide prison service [21], 27 statewide male prisons [30], five country wide studies across male and female prison settings [23,28,29,31,32] and one based on four statewide correctional facilities for men [26]. One study recruited service users with a recent history of incarceration through an AIDS service organization [22] and another recruited older adults who were either released from or were currently serving time in a state prison [27]. Two studies were based in a single male adult prison [24,25]. Of note is that out of the twelve papers meeting the inclusion criteria, nine concentrated on the psychosocial experiences of trans people in prison and predominantly the trans-feminine experience. Whilst sexual minority and gender minority prisoners face some shared and distinct issues in prisons, the LGBTQ+ umbrella term is limiting and can alienate the specific issues of the subgroups in this term. This issue is addressed in the limitations section of this review. All of the studies used qualitative research methods that explored and documented the experiences and perceptions of LGBTQ+ people in prison. The study characteristics are presented in Table 3.

### 3.3. Thematic Analysis

**Figure 2:**
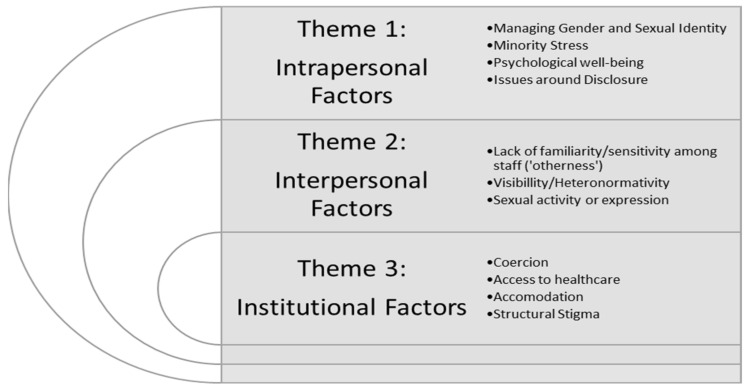
Thematic map.

Following the systematic analysis of the studies, three major themes were identified, represented in Figure 2.

#### 3.3.1. Theme One: Intrapersonal Factors

LGBTQ+ people are over-represented in the criminal justice system. Despite this, their distinct physical, psychological and social needs remain hidden or overlooked [21,22,23]. Sexual orientation, sexual and gender identity are terms that have only recently received attention in criminal justice systems and the experiences of LGBTQ+ people while incarcerated [24,25]. There are many reasons that LGBTQ+ people may choose not to disclose their gender identity or sexual orientation whilst in prison due to the threat of transphobia, homophobia and biphobia [23,26,27]. Many people feel discriminated against, stigmatized, threatened and may encounter physical or sexual violence or abuse [21,22,25]. The process of ‘coming out’, ‘being out’ and ‘staying out’ was problematic and depended very much on the context and the ‘audience.’ Disclosure, if evident, was measured and carefully and safely revealed. This often led to hypervigilance and increased stress and anxiety [23,28]. Some people feared becoming even more marginalized, ostracized and estranged from their families, friends and communities [25,29].

Prisons remain heteronormative environments and are not conducive or responsive to the issues and concerns of LGBTQ+ prisoners [29,30,31]. As a consequence, the psychosocial needs of this population are left ignored, hidden and unmet. For instance, there are significantly higher incidences of depression, anxiety and suicidality compared with the non-LGBTQ+ population [22,23]. There is evidence that homophobia is ubiquitous in men’s prisons as a result of a ‘corrections culture’ of pronounced masculinity and rigid hierarchy, often supported through violent means. Significant numbers of male prisoners can demonstrate higher homophobic disapproval than those in the general population [25]. These attitudes and behaviours can also be mirrored among staff in many prisons. This can result in the degradation, devaluing and victimization of LGBTQ+ prisoners who are not perceived as being ‘masculine enough’ [23,28].

This can lead to increased ‘invisibility’ and the further marginalization of non-heterosexual or non-binary prisoners. Conversely, trans women may not be perceived as ‘feminine enough’ or effectively ‘pass’ in a prison as men. Many appear to seek for positive affirmation of their womanly characteristics. They strive for a ‘ladylike’ ideal and to be seen as close enough to the ‘real girl’ in order to achieve their accomplishment of gender in prisons for men [30]. Where female identity is ignored, it can lead to increased issues and concerns regarding physical, psychological and gender-transitioning healthcare needs [21,22,28,32].

LGBTQ+ people have had to find ways of managing their sexual and gender identities despite the manifestations and impact of minority stress in the prison setting. Increased interest is being paid to the unique experiences of gay and bisexual men, before, during and after incarceration. In one study, men had to learn and apply identity management strategies to help them cope with the consequences of the prevailing anti- gay and bisexual attitudes in prison [25].

#### 3.3.2. Theme Two: Interpersonal Factors

The factors that influence the relationships of LGBTQ+ inmates to both other inmates and staff was the focus of many of the articles in this review [21,23,27,29,30,32]. There is a global consensus that the risk of harassment, abuse and violence is a dominant issue of concern for LGBTQ+ prisoners [33,34]. It is also recognized that the regulation of gender identity is a means of social control within the prison setting and as such, underlies many of the interactions between prisoners and staff [35]. In particular, the inmates of Rosenberg and Oswin’s study [32] described how masculine norms are imposed in prison through disciplinary punishment and/or social repercussions. This includes but is not limited to verbal harassment, isolation, and physical assault. The study conducted by Yap et al. [23] suggests that the prison environment can apply significant pressure on gay and bisexual men on the management of their sexual identities and disclosure of their sexuality in prison. This has to be negotiated by prisoners vis-à-vis the backdrop of heteronormative values and hyper-masculine culture [23].

In terms of relationships with other inmates, the study undertaken by Wilson et al. [28] attempts to develop the distinct sexual experiences of trans women in prison and highlights the complexity of these experiences, that are more nuanced than previously documented accounts of sexual assault. Their findings challenge the dominant narrative of prison rape and instead highlight the participant’s attempts to negotiate opposing categories of ‘vulnerability’ and ‘power/opportunity’. Trans prisoners in this study pursue a heteronormative femininity that provides agency, pleasure and protection.

Within the microcosm of prison culture, staff were often perceived by LGBTQ+ inmates as lacking in familiarity in gender or sexuality specific issues and as a result, were experienced as insensitive or oppressive. In the study conducted by Jaffer et al. [21] exploring transgender inmate’s experiences, the need for prison staff to receive specific transgender healthcare training was recognized. Nearly all participants in this study stated that they felt there was a lack of familiarity and sensitivity to the concerns of transgender individuals among staff. Based on these findings, LGBT trainings were conducted across 12 prison clinics and three months after the training, inmate complaints were reduced by over 50%.

#### 3.3.3. Theme Three: Institutional Factors

Most people in prison are forced to navigate a rigid and hyper-masculine environment where the focus is on adherence to rules, norms and conformity [34]. The evidence arising from the current review highlights that within the prison system, LGBTQ+ people are frequently ‘othered’ and as a result their healthcare and other needs can be neglected or appropriate care and support withheld [22,26,31]. Within institutional settings, discrimination against LGBTQ+ people occur along a continuum ranging from invisibility to overt behaviours including verbal and physical abuse [31]. In the study conducted by White-Hughto et al. exploring the experiences of 20 transgender women, participants described how their feminine identity was not recognized and the policies of the institution acted as a form of structural stigma. This resulted in the creation and reinforcement of a gender binary and restrictions in access to healthcare [31]. Although reasons behind this varied from institutional to interpersonal, the result was insufficient access to physical, mental and gender transition-related healthcare, negatively impacting upon participants’ health while incarcerated.

In the study undertaken by Wilson et al. [28] investigating the experiences of transgender women, there was a call to acknowledge the more nuanced sexual experiences reported. This includes the less visible sexual harassment and coercion which can have a detrimental impact on trans women’s psychological well-being [28].The study conducted by Rosenberg and Oswin corroborates these findings, evidencing that transgender women endure harsh conditions of confinement within the hyper-masculine and heteronormative environment of the US prison system [32]. Of particular note also for transgender people in prisons are the difficulties accessing appropriate healthcare support for their transition. The lack of response by the institution is compounded by ongoing misunderstanding, discrimination and the downplaying of sexual identities. For example, transgender women are typically housed in facilities that are segregated according to their genitalia rather than their chosen sexual identity [30].

In another study undertaken by McCauley et al. focusing on transgender women of color, participants experienced varying degrees of abuse and harassment either through solitary confinement or lack of access to hormone treatment, resulting in significant mental health issues [24]. The authors advocate for educational programs for all prison staff specifically on the importance of medications for gender transition and access to mental healthcare [24]. In another study of transgender women of color who were HIV positive [22], participants revealed that many and varied forms of stigma experienced that resulted in a type of institutional coercion. This had the effect of suppression of their gender identity and the inappropriate use of solitary confinement, both of which led to increased psychological distress during their time in prison [22].

## 4. Discussion

The evidence presented in this review highlights that when in prison, many LGBTQ+ people experience significant discrimination that impacts on their health and well-being. Yet, despite the growing recognition of the need to identify and protect potentially vulnerable groups in prison settings, there is a need to more fully understand those of LGBTQ+ people. This review therefore aims to fill that gap by identifying their unique views and experiences and the actions required to address their intervention, care and support needs. As pointed out earlier, the majority of papers in this review concentrated on the trans feminine experience in prison settings. Whilst it is not the intent of this review to generalize these experiences within and across the LGBTQ+ umbrella, the small number of papers that concentrated on gay, lesbian or bisexual populations supported the findings discussed here.

Despite the high numbers of LGBTQ+ people in prison, heteronormativity prevails with fears of homophobia, biphobia and transphobia linked to physical and sexual assaults. Highly heteronormative institutions such as prisons are based on clear gender divisions, and are thus more homophobic and transphobic spaces than the societies in which they exist. This type of environment may prevent or discourage LGBTQ+ people from identifying themselves to other prisoners, staff or indeed to researchers. Therefore, the decision to reveal one’s sexual identity is an issue requiring careful consideration due to the multiple potential consequences. The situation is further compounded by the lack of awareness of behalf of prison staff of the specific needs and concerns of LGBTQ+ people and how to respond appropriately. The evidence therefore points to the need to specific training and education for prison staff on the needs of LGBTQ+ people and specific issues relating to the subpopulations of this group. Failure to recognize and address the specific and distinct needs of LGBTQ+ populations lead to their further marginalization and discrimination which impacts on their often already poor health. With the growing evidence-base and recognition of the scope of the health inequalities and health needs experienced by many LGBTQ+ people more generally, there is a need and an opportunity to ensure they are recognized, identified and addressed in the prison setting. There are therefore important policy, practice and education and development implications arising from the current review, with developments necessary to address the specific needs and concerns of LGBTQ+ people in prison. Future research opportunities have been identified that will add significantly to the body of evidence that may further the development of appropriate health interventions and supports to this population.

### 4.1. Policy

Despite claims of equality and Human Rights for all, there continues to be many countries across the world where being LGBTQ+ remains a criminal offence involving lengthy prison sentences or in some cases, death [36]. This is notwithstanding, the United Nation position on LGBTQ+ fundamental rights and the need to have in place LGBTQ+ specific policies, including ones that respond to the needs of those in prisons [37]. Such policies are necessary as LGBTQ+ people are overrepresented within prison populations and have specific psychosocial concerns and needs that require to be identified and addressed [38]. Arising from this is the need to recognize and respond to the specific and distinct needs of the sub-populations [39]. This is important from a policy perspective as the needs of gay men, lesbians, trans are not the same and need to be reflected in both prison policies and health policies to ensure their needs are properly identified and addressed [34]. There is a need for appropriate health policies to ensure that prison health providers have systems and processes to identify, assess and have in place individualized care plans, thereby responding to and further minimising the already poor health outcomes experienced by LGBTQ+ people [3,4,34].

### 4.2. Education and Practice Development

A recurring theme across the studies included in this review is the need for education to develop and improve the knowledge and skills of professionals working in prisons concerning the needs of LGBTQ+ people and across the sub populations. Additionally, a new area requiring a specific education focus are the responses required to address the changing demographic of the LGBTQ+ population in prison including LGBTQ+ youth, seniors and trans people, necessary due to their specific health concerns [34,40,41,42]. This is line with some of the recommendations of the Prison Rape Elimination Act in the US [15]. This specifies that penal facilities must provide LGBT education to personnel in order to comply with federal policy. To respond appropriately at an international level however, there is a need for undergraduate and postgraduate education programmes for doctors, nurses, social works, clinical psychologists and others on the specific care and support needs of LGBTQ+ people in general and more specifically when in prison [43]. Education and practice developments are necessary to ensure continuity of health care interventions and treatments such as mental treatment and interventions and hormone therapy for trans people [37,44,45]. There are also shared learning opportunities at postgraduate level across health and social care professions to enhance and improve knowledge and understanding of the needs and concerns of LGBTQ+ people when in prison. Continuing professional development learning opportunities also need to be in place between prison staff and health professionals to develop knowledge and understanding of the needs of LGBTQ+ people in prison and thereby enabling appropriate responses [3,4,15,21,34,36,37].

### 4.3. Practice

This systematic review has highlighted that the needs of LGBTQ+ people when in prison continue to be met within a heteronormative and non-gender affirmative context [23,33]. The evidence within the review suggests that the distinct needs of LGBTQ+ people are often ignored and as a consequence remain hidden, ignored or poorly understood [24,25,28,29]. Health professionals working in prison health require a wide range of knowledge and skills to meet the diverse physical, mental health and behavioural needs of prisoners, including LGBTQ+ people. This systematic review has identified experiences and concerns regarding abuse, assaults, exploitation, rape, prejudice and discrimination leading to hyper-vigilance and *minority stress*, impacting negatively on access to healthcare and health outcomes [22,23,24,25]. Therefore, professionals working in prison health have important roles to play in recognizing and supporting LGBTQ+ people to ensure that appropriate assessment, treatments, interventions and supports are in place. This is important as the needs of the LGBTQ+ subpopulations differ and require specific responses [3,4,13,21]. Professionals working in prison health have a responsibility to ensure that they have the required knowledge, skills, attitudes and values, necessary to respond to the needs of LGBTQ+ people in prison, ensuring access to healthcare that is free from fear of prejudice, discrimination and abuse. For this to be a reality, it is necessary for care and support to be informed by current research evidence and best practice thereby responding appropriately to the specific needs of LGBTQ+ people in prison. Further qualitative research drawing on the views and experiences of LGBTQ+ people will help to develop the understanding of their specific care and support needs and the extent to which they are being identified and addressed.

## 5. Strengths and Limitations

This is the first systematic review, that the authors are aware of, to focus on the specific views and experiences of LGBTQ+ people in relation to the care and support when in prison. The review provides important directions for policy, education, practice and future education initiatives to promote the provision of evidence-based health care and support that is responsive, sensitive and appropriate to the needs of LGBTQ+ people when in prison. Several limitations have been identified from this review. The parameters of this review detail issues of ‘psychosocial concern’; whilst this is a legitimate focus given the multi-layered issues faced by this population in prison, it is not conclusive of all LGBTQ+ experiences. The majority of the studies included in this review were undertaken in the United States of America, with limited research undertaken in Europe, Africa and South America. The relatively unique context of imprisonment in the US, both in terms of scale and the prison environment means that there is a need to be cautious of generalising these findings. There is a considerable disparity in the different types of carceral settings both nationally and internationally. Therefore, it is important to recognize that the views and experiences of LGBTQ+ people in prison may differ considerably, both positively and negatively. For many of the papers included in this review, there was a lack of clarity around comparative factors of the prison trans populations. Ideally in future research, all comparisons should be disaggregated by both sex assigned at birth and current gender identity. Finally, the majority of the papers in this review (n = 9) concerned trans prisoner experiences. As such, the review allows for more substantive analysis of the needs and experiences of trans prisoners than other groups within the LGBTQ+ umbrella. It is of vital importance that the unique challenges experienced by the other sub-groups of this population are recognized in future studies. Failure to do this would mirror the inaccurate conceptualization of researchers and society at large conflating LGBTQ+ subgroups into a single population. The authors sought to be rigorous in the review process with recognizing the potential for subjectivity and bias.

## 6. Future Research

This systematic review clearly evidences that despite legislation and policy recognizing their needs, many LGBTQ+ people continue to experience significant discrimination and health disparities in life in general and specifically when in prison. In relation to their health disparities, further research is required to identify and reduce the factors that lead to institutional discrimination experienced by LGBTQ+ people when in prison. Lack of knowledge of the distinct needs of LGBTQ+ people is an area requiring a research focus to enable the development, implementation impact and evaluation of education programmes. The majority of papers that met the inclusion criteria of this review were of trans prisoner experiences. There remains generally a lack of literature on sexual minority prisoners and it is key to further research. There is a need therefore for further research to improve the understandings of the specific needs of sexual minorities within the LGBTQ+ population when in prison to ensure their specific needs are fully understood and evidence-based supports and interventions provided that improves the standard of their care. The majority of the studies included in this review were single centre and cross-sectional, with no longitudinal designs. There is scope therefore for both national and international multicentre and longitudinal studies that identifies the needs of LGBTQ+ people in prison over time and how effectively or not they are identified and met and the impact on their health outcomes.

## 7. Conclusions

LGBTQ+ people are disproportionately represented in correction facilities. The findings of this review show the ways in which the LGBTQ+ population in prison find themselves in a rigid, restrictive, heteronormative and hyper-masculine environment where the focus is on adherence to rules, norms and conformity. Within the binary structure of the prison system, the needs of LGBTQ+ people tend to be largely ignored, leading to feelings of helplessness, isolation and at times, humiliation. Whilst there are promising efforts to improve the prison environment, individual and institutional barriers exist and conspire to make being LGBTQ+ in prison a challenging experience.

The findings arising from this review highlight the many challenges that continue to be the reality for many LGBTQ+ people when in prison. LGBTQ+ people have highly variable experiences when in prison with many of their health needs remaining unidentified and unmet, potentially impacting negatively on their health and well-being. Many LGBTQ+ people in prison face a myriad of complex issues to consider, such as the decision or not to disclose their sexual identity for fear of discrimination, abuse and violence. Even when their LGBTQ+ sexual identity is disclosed, it does not follow that the necessary services and supports will be provided and, in some situations, they appear to be absent. This concern is particularly important in relation to LGBTQ+ youth in prison who may be the subject of further exploitation and harm. The LGBTQ+ population is evolving and changing across the age continuum with a need to recognize and respond to older LGBTQ+ seniors in prison who may present with health needs associated ageing, some of which may be attributed to with sexual health needs such as HIV. There is continued ignorance and prejudice directed at many LGBTQ+ people when in prison that an increased focus on education may gradually start to erode and address. However, from the available evidence, education focusing on the needs of LGBTQ+ people in prison appears to be lacking and inconsistent. There is therefore a need to develop consistent approaches to the inclusion of LGBTQ+ needs and concerns within professional undergraduate, post-registration programmes for doctors, nurses, social works, clinical psychologists and others and within CPD education programmes within prisons. While there is a much-needed evolving research evidence base on the specific views and experiences of LGBTQ+ people in prison, future studies need to also focus on the impact and outcomes on the care and support experiences within prisons. Without such attention, the health disparities experienced by LGBTQ+ people in prison will only continue to grow.

## Figures and Tables

**Table 1:** Search results on 5th April 2021 *.

Search Code	Query	PsycINFO	MEDLINE	CINAHL
S1	LGBT * OR gay OR homosexual OR ‘sexual minority’ OR transgender OR bisexual OR lesbian	15,436	18,233	15,434
S2	‘mental health’ OR psychosocial	89,446	46,588	17,001
S3	prisons OR jail OR penitentiary OR correctional OR ‘penal institution’ or lockup OR prisoner or inmate OR convict OR criminal OR offender OR incarcerated	55,302	36,734	8166
S4	opinions OR views OR perceptions OR experiences OR qualitative	637,325	908,616	21,861
S5	S1 AND S2 AND S3 AND S4	281	89	59

* Limiters: academic peer reviewed papers, written in English, published since 2010.

**Table 2:** CASP quality scores.

CASP Criteria	Harvey et al. (2021)	Hochdorn et al. (2018)	Jaffer et al. (2016)	Janness and Fenster-Maker (2014)	Kilty (2020)	Maashi et al. (2016)	McCauley et al. (2018)	Rosenberg and Oswin (2015)	Sumner and Sexton (2016)	White-Hughto et al. (2018)	Wilson et al. (2017)	Yap et al. (2020)
1. Clear statement of aims	Y	Y	Y	CT	Y	Y	Y	Y	Y	Y	Y	Y
2. Appropriate methodology	Y	Y	Y	Y	Y	Y	Y	Y	Y	Y	Y	Y
3. Appropriate research design	Y	Y	Y	Y	Y	Y	Y	Y	Y	Y	Y	Y
4. Appropriate recruitment strategy	Y	Y	Y	Y	Y	Y	Y	Y	Y	Y	Y	Y
5. Appropriate data collection methods	Y	Y	Y	Y	Y	Y	Y	Y	Y	Y	Y	Y
6. Research relationships considered	CT	CT	N	N	Y	CT	Y	CT	CT	Y	Y	CT
7. Consider ethical issues	Y	Y	Y	N	Y	Y	CT	CT	CT	Y	CT	CT
8. Rigorous analysis	Y	Y	CT	CT	Y	Y	Y	Y	Y	Y	Y	Y
9. Clear findings	Y	Y	Y	Y	Y	Y	Y	Y	Y	Y	Y	Y
10. Value of the research	Y	Y	Y	Y	Y	Y	Y	Y	Y	Y	Y	Y

Y: Yes; N: No; CT: Can’t tell.

**Table 3:** Papers included in the review (n = 12).

Citation and Country	Aim	Sample	Methods	Main Results	Recommendations
Harvey et al. (2021) USA [25]	To examine previously incarcerated gay and bisexual male (GBM) experiences of minority stress and management of their sexual identity.	Formerly incarcerated GBM in New York City (n = 20). Mean age 40.2 (±12.4)	Semi-structured, in-depth interviews.	Findings document the ways in which this population manages their sexual identities in the context of minority stress alongside the associated psychosocial health risks. Themes include minority stress: (1) as catalyzing incarceration-related experiences, (2) as motiving identity management techniques and (3) as a determinant to re-entry support and sexual expression after incarceration.	Recommendations are for changes to public health policy and practice. These changes will better serve the needs of incarcerated GBM and will inform practice that aims to prevent incarceration in the first place.
Hochdorn et al. (2018) Italy/Brazil [29]	To investigate how the discursive positioning among the ‘Self’ and the ‘Other’ might promote the internalization of positive and/or negative attitudes toward the self in trans women.	Transgender women detained in prison contexts in Italy and Brazil. Aged 24–51 years	In-depth interviews	The findings demonstrated language differences amongst transgender inmates in Brazilian and Italian samples. Additionally, in Brazil, transgender women assumed masculine-driven behavior due to a common imprisonment with cis-gender men. Transgender women in Italy however are detained in protected sections, where they are allowed to wear female clothing and continue hormonal treatments. Finally, transgender inmates in Italy suffered more violence in a female sector when compared to exclusively male jails.	The needs of transgender people should receive special attention as they vary greatly to their cis gender inmates. Psychological counseling with transgender women should pay particular attention to the psycho-social issues of this population.
Jaffer et al. (2016) USA [21]	To review and evaluate the provision of care for transgender people in the New York City prison system.	Transgender people housed in jail facilities (n = 27) Age range—n/a	A brief in-person survey	The dominant concern of transgender people in prison was their inability to obtain hormone therapy. Almost all participants felt there was a lack of familiarity and sensitivity to their specific health and other concerns.	Opportunities exist to deliver dedicated services to the transgender population in prison. Participants recommended hiring clinical staff with transgender experience or designating qualified transgender healthcare providers. All particiapants emphasized the need for specific transgender housing.
Jenness and Fenstermaker (2014) USA [30]	To explore how gender is accomplished by trans prisoners in prisons for men.	Transgender inmates (n = 315). Age range—n/a	Semi-structured interviews	In prison, transgender people engage in behaviors that constitute what the authors refer to as the pursuit of gender authenticity. There exists a gender order for participants that underpins prison life for transgender inmates.	Further research necessary looking specifically at transgender women in order to understand more about the context of living as transgneder in prisons.
Kilty (2020) USA [22]	To explore how stigma emerges in the prison environment and to explore the ways that HIV and transgender stigma are linked to harmful practices.	Black HIV-positive transgender women (n = 10). Age range—n/a	Interviews, face-to-face.	Participants revealed that the many different forms of stigma resulted in a type of coercive practice that resulted in the suppression of their gender identity. They also spoke of the inappropriate use of solitary confinement and their expereinces of often being denied access to HIV medication and hormone replacement therapies (HRT).	In order to help curb transgender and HIV stigma and discrimination it is essential to significantly improve correctional staff members and health care providers knowledge about HIV and transgender issues. Mandatory transgender and HIV education classes and sensitivity training would help to build cultural and clinical competence.
Maashi et al. (2016) USA [27]	To explore the experiences of formerly incarcerated LGBT elders before, during, and after prison.	LGBT elders (n = 10). Aged 50–65 years.	Focus groups and individual interviews	A core theme that emerged concerned LGBT elders ongoing coming-out process that is concurrently being managed via multiple stigmatized identities. These findings increase our awareness of an often neglected population of LGBT who are older and in prison.	Formerly incarcerated LGBT elders should be included in future recommendations for services and policy reform in carceral settings.
McCauley et al. (2018) USA [28]	To document the health-related experiences and needs of transgender women of color in prison.	Transgender women of color (n = 10) Age range—n/a	Semi-structured interviews	Participants experienced high levels of abuse and harassment. This led to mental health issues which were exacerbated by the lack of access to hormone treatments.	Policy changes necessary to address housing issues, and to improve access to healthcare for transgender women in prison. Training is required for prison staff to better understand the unique needs and experiences of transgender people.
Rosenberg and Oswin (2015) USA [32]	To examine the experiences of incarcerated transgender people.	Trans feminine inmates (n = 23) Aged 19–50 years.	In-depth questionnaires	Participants experienced harsh conditions of confinement. Part of the diffculty with carceration for this population is having to cope with hypermasculine and heteronormative prison environment.	None identified.
Sumner and Sexton (2016) USA [26]	To examine the “dilemma of difference” transgender prisoners face within a sex-segregated prison system.	Transgender prisoners (n = 10), prisoners (n = 27) and prison staff (n = 20) Mean Prisoner Age-41 Years	In-depth qualitative interviews and focus groups.	Transgender prisoners are and should be treated like everyone else, despite their unique situations. Themes addressed included the differences and meaning of being transgnder in prison. The consequences of these differences are also addressed.	Need for provision of gender specific clothing, housing assignments, and treatment. In order to esure this, the institution must understand the point of difference for transgender prisoners in the context of gender as opposed to sexuality.
White-Hughto et al. (2018) USA [31]	To explore the healthcare experiences and interactions with correctional healthcare providers of incarcerated transgender women.	Transgender Women (n = 20) Mean age 36.9 years (SD ¼ 10.0)	Semi-structured interviews.	Participants described an institutional culture in which their feminine identity was not recognized. They also described the ways in which prison policies acted as a form of structural stigma. Some participants attributed healthcare barriers to bias whilst others understood it as provider’s limited knowledge of transgender issues. These barriers to appropriate physical, mental, and gender transition-related healthcare negatively impacted participants’ health while incarcerated	Delivery of healthcare to incarcerated transgender individuals is under researched. Access to gender affirmative care for incarcerated transgender communities can be achived throught educational and policy interventions.
Wilson et al. (2017) Australia [28]	To examine the sexual experiences of trans women in men’s prisons specifically addressing sexual safety.	Transgender Women (n = 7). Aged 20 to 47 years.	Semi-structured interviews.	Whilst there were some rape experiences described by particpants, accounts of sexual activity were not always physically violent and issues of consent were not always clearly defined.	There is a need to look at ways to prevent the incarceration of trans women. In the absence of this, recomendations are needed to explore ways in which trans women can be better supported in the prison setting.
Yap et al. (2020) Australia [23]	To explore the concept of coming out in prison.	Prisoners and one ex-prisoner who self-identified as gay, homosexual or bisexual men (n = 13). Aged 20–59 years.	In-depth interviews	Respondents were required to continuously manage their sexual identities and disclosure to different audiences while incarcerated. Findings suggest that the heteronormative prison environment and its’ consequences, apply considerable pressure on gay and bisexual men, around mangaing the disclosure of their sexual identity.	None identified.

## Data Availability

No new data were created or analyzed in this study. Data sharing is not applicable to this article.
